# Infectious Prions Accumulate to High Levels in Non Proliferative C2C12 Myotubes

**DOI:** 10.1371/journal.ppat.1003755

**Published:** 2013-11-07

**Authors:** Allen Herbst, Pamela Banser, Camilo Duque Velasquez, Charles E. Mays, Valerie L. Sim, David Westaway, Judd M. Aiken, Debbie McKenzie

**Affiliations:** 1 Centre for Prions and Protein Folding Diseases, University of Alberta, Edmonton, Alberta, Canada; 2 Department of Agriculture, Food and Nutritional Sciences, University of Alberta, Edmonton, Alberta, Canada; 3 Department of Biological Sciences, University of Alberta, Edmonton, Alberta, Canada; 4 Division of Neuroscience, Department of Medicine, University of Alberta, Edmonton, Alberta, Canada; University of Edinburgh, United Kingdom

## Abstract

Prion diseases are driven by the strain-specific, template-dependent transconformation of the normal cellular prion protein (PrP^C^) into a disease specific isoform PrP^Sc^. Cell culture models of prion infection generally use replicating cells resulting in lower levels of prion accumulation compared to animals. Using non-replicating cells allows the accumulation of higher levels of PrP^Sc^ and, thus, greater amounts of infectivity. Here, we infect non-proliferating muscle fiber myotube cultures prepared from differentiated myoblasts. We demonstrate that prion-infected myotubes generate substantial amounts of PrP^Sc^ and that the level of infectivity produced in these post-mitotic cells, 10^5.5^ L.D._50_/mg of total protein, approaches that observed *in vivo*. Exposure of the myotubes to different mouse-adapted agents demonstrates strain-specific replication of infectious agents. Mouse-derived myotubes could not be infected with hamster prions suggesting that the species barrier effect is intact. We suggest that non-proliferating myotubes will be a valuable model system for generating infectious prions and for screening compounds for anti-prion activity.

## Author Summary

This manuscript describes the generation of a new cell culture system to study the replication of infectious prions. While numerous cell lines exist that can replicate prions, these systems are usually based upon proliferating cells. As mammalian cell cultures double approximately every day, prions established in the culture must also, at least, double to be maintained. This is problematic, however, as prions replicate relatively slowly and cell replication may outpace prion replication. In fact, many cell culture systems do not replicate prions and those that do often do not replicate all strains of prions. Here we describe the use of differentiated non-proliferative muscle cells to replicate prions without the interfering effect of cell division. We observed that prions accumulate to very high levels in this muscle cell culture with infectivity approaching that observed in animals.

## Introduction

Prions are the etiological agents responsible for the transmissible spongiform encephalopathies. These neurodegenerative diseases affect mammals, are inevitably fatal, and are always associated with the accumulation of a specific post-translationally modified isoform of a normal host glycoprotein, PrP^C^. This abnormal conformation, the PrP^Sc^ isoform, differs from PrP^C^ structurally, resulting in dramatic functional consequences. While PrP^C^ is readily degraded by proteases, soluble in detergent and rich in alpha-helical structure, PrP^Sc^ is typically characterized by resistance to proteinase K (PK) digestion, detergent insolubility, amyloid formation and β-sheet structure. *In vivo* the disease-specific isoform of the prion protein (PrP^Sc^) accumulates to high levels, a process that is marked by a progressive neurodegeneration that is always fatal as well as the generation of hundreds of millions of lethal doses of transmissible prions.

Experimentally, prion infections are typically performed in rodents: wild type mice, transgenic mice or hamsters. Incubation periods are generally “short” (compared to prion infections of cervids, sheep or cattle), ranging from two months in hamsters and certain transgenic mouse lines to greater than a year for other mouse strains and agent strain combinations. Brain infectivity levels are extraordinarily high at clinical stage, 10^9^ 50% lethal doses (LD_50_) in end stage hamster brain and 10^8^ LD_50_ in mouse models.

*In vitro* models of prion replication have been established by incubating infectious brain homogenates with various susceptible cell lines, most of neuronal origin, and all expressing PrP^C^, obligatory as a source for PrP^Sc^ generation. Typically, dividing cells are exposed to brain homogenates, derived from infected mice, and the cells are then serially passaged until the inoculum is diluted out. PrP^Sc^ accumulates to a steady state determined by the accumulative effect of prion replication, the dilutive effect of cell division and subsequent passaging, prion secretion into the media [1], [2] and prion degradation [3]–[6]. *In vitro* replication of prions has been observed in numerous cell types including scrapie mouse brain cells [7]–[9], fibroblasts [10], [11], epithelia [12], glia [13], [14], microglia [15], PC12 [16], Schwann cells [17], hypothalmic neurons [18] and neuron-like cells [19]. By far, however, the most widely used cells for *in vitro* replication of prions are mouse Neuro-2a (N2a), neuroblastoma cells [13], [20]–[22]. Prion infection of cell cultures typically results in relatively low levels of PrP^Sc^ and infectivity being generated. In the N2a cells, the level of infectivity is very low, ∼3×10^3^ LD_50_ per 1×10^7^ cells [20], [21]. When infected neuroblastoma cell lines are subcloned and highly susceptible sublines are isolated however, the infectivity can increase to ∼2×10^4^ LD_50_ per 1×10^7^ cells [23]. Alternatively, highly susceptible N2a sublines can be isolated and subsequently infected [24], allowing for cognate uninfected cells to be propagated as controls.

One difficulty in generating and maintaining *in vitro* cultures of prion infection is that the infectivity levels are low and some species and strain combinations do not result in infection or stable infection [24]–[27]. Cell lines that divide rapidly tend not to support prion replication, presumably due to the dilutive effect of cell replication [28]. One potential means of overcoming these effects is the use of post-mitotic, differentiated cells for studies of prion replication. Murine-derived C2C12 myoblast cells [29] provide an intriguing possibility as myoblasts are proliferative but following serum deprivation, terminally differentiate into post-mitotic myotubes, a syncytium of fused myoblasts. Muscle expresses relatively high levels of PrP^C^ [30],which promotes muscle regeneration *in vivo* [31], and can harbour and replicate prion infectivity [32], [33].

We report that differentiated non-proliferative myotube cultures can replicate prions to surprisingly high levels. Previous work with this cell culture system only observed infection, with 22L prions, when C2C12 cells where co-cultured with susceptible neuroblastoma cell lines [34]. Our studies have focussed on the infection of myotubes, not myoblasts, an approach that may prove useful as the terminally differentiated cells do not divide, removing the dilutive effect of passage in assays of scrapie replication. This system more closely mimics the *in vivo* situation, where a less dynamic population of cells accumulates PrP^Sc^.

## Results

### Expression of PrP^C^ in terminally differentiated myotubes

**Figure 1:**
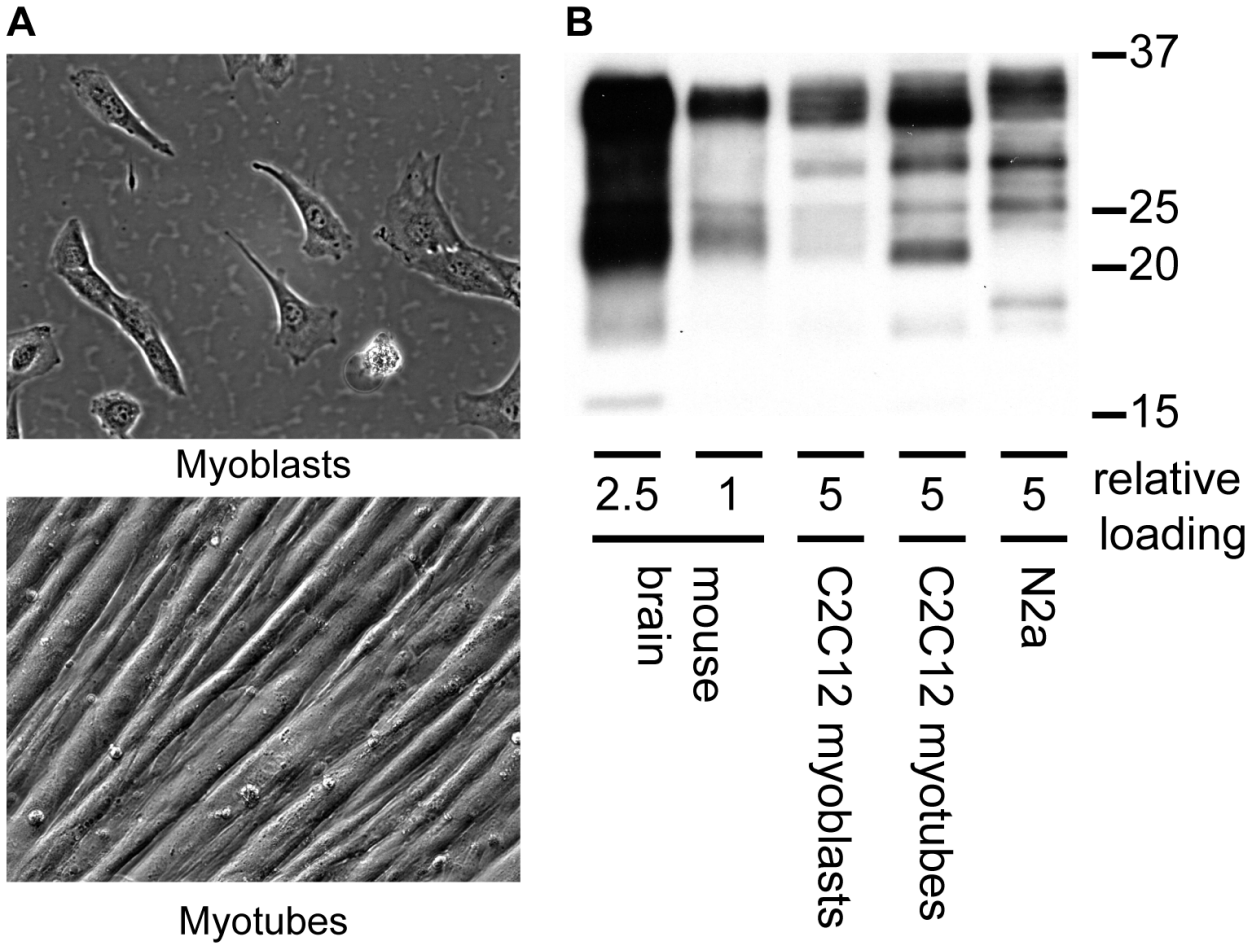
Expression of PrP^c^ in C2C12 myoblasts and myotubes. (A) Morphology of C2C12 myoblasts and myotubes (B) Western blot comparison of PrP^C^ expression from mouse brain homogenate, C2C12 myoblasts and myotubes and N2a neuroblastoma cells. Relative loads are based on protein concentration of the brain homogenate (1.78 µg, 0.68 µg protein) and cell lysates (3.4 µg C2C12 myoblasts, myotubes and N2a). Anti-PrP antibody SAF 83 was used.

Proliferative myoblasts are capable of undergoing terminal differentiation into muscle fiber-like myotubes (Figure 1a). Spontaneous differentiation occurs at high cell density and after serum withdrawal. Fully differentiated myotubes are multinucleated, contain sarcomeres and can contract. Monolayers of myotubes can remain intact for weeks. Importantly, as a cell culture system for PrP^Sc^ replication, myotubes express approximately one fifth as much normal prion protein (PrP^C^) as brain normalized per mg protein and an equivalent amount to N2a neuroblastoma cells (Figure 1b) though N2a cells are known to be variable in their characteristics [35]. Slightly higher levels of PrP^C^ are consistently observed in C2C12 myotubes compared to myoblasts. PrP^C^ expressed in myoblasts is predominately di-glycosylated.

### Myotubes accumulate PrP^Sc^ whereas myoblasts do not

**Figure 2:**
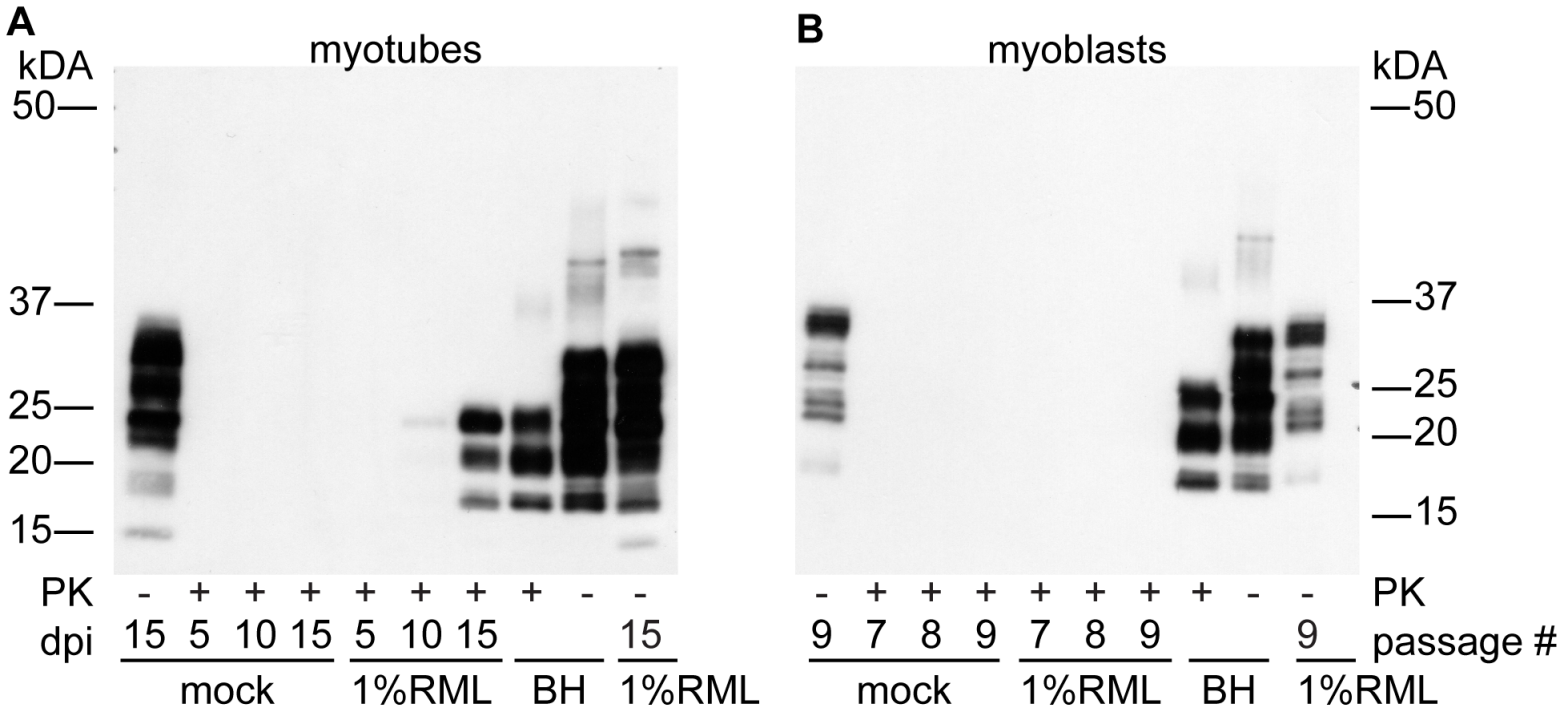
PrP^Sc^ replicates in myotubes. (A) Time course of PrP^Sc^ accumulation. Confluent myotubes were infected by incubation of cells in media spiked with 100 µL of 1% RML brain homogenate or mock infected with normal brain homogenate overnight. Media was changed daily. After 5, 10 or 15 days in culture, cells were lysed and analyzed for PK-resistant PrP. (B) Myoblasts were infected by incubating in media spiked with 100 µL of 1% RML brain homogenate or normal brain homogenate. Cells were analyzed after passages 7, 8 and 9 which correspond to myotube days 11, 13 and 15 respectively and no PrP^Sc^ was observed. For both panels (A,B), 7.5 µg of protein was loaded in PK- lanes. 15 µg of protein equivalent was loaded in lanes treated with PK. 10 µl of 0.15% BH was loaded ∼11.25 µg protein. Anti-PrP antibody SAF 83 was used.

To examine the replicative potential of prions in a terminally differentiated muscle cell line and contrast them with non-differentiated replicative muscle precursor cells, we exposed both C2C12 myoblast and myotube cultures to RML prions (Figure 2). C2C12 cells were propagated and expanded as myoblasts, seeded into individual wells or flasks, grown to confluence and differentiated into a layer of myotubes upon serum withdrawal. Myotubes can easily be infected with brain homogenates by diluting infected brain homogenate into the media. Myoblast cultures were infected as sub-confluent proliferating cultures, splitting as necessary to prevent differentiation. By passage nine, fifteen days after infection, accumulation of PrP^Sc^ was not detectable in myoblast cultures (Figure 2b) consistent with the results of Dlakic et al. 2007 [34]. The infection of differentiated myotubes gave a very different result. We routinely observed accumulation of newly generated proteinase K resistant PrP^Sc^ by 10 days post-infection in myotubes (Figure 2a). Levels of PrP^Sc^ subsequently increased with time.

### Myotube-generated PrP^Sc^ is highly infectious

**Figure 3:**
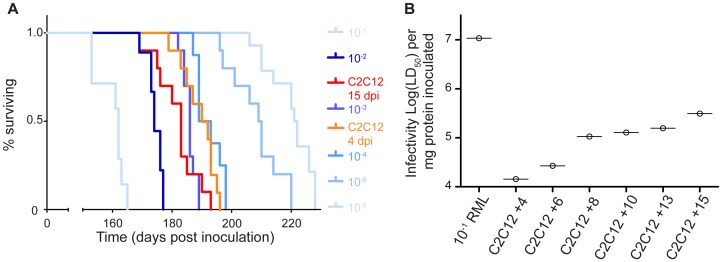
C2C12 myotubes replicate prions to high levels of infectivity. (A) C2C12 myotubes infected with RML were collected at 4 or 15 days post infection by scraping in PBS, centrifuging and resuspending in 500 µL of water. 30 µL of this cell material was inoculated intracranially into weanling C57Bl/6 mice. A statistically significant difference in incubation period was observed between 4 and 15 days indicative of prion replication p<0.05. Parallel inoculation of serial 10-fold dilutions of RML brain homogenates were performed. (B) Plot of infectivity in each inocula standardized per gram of protein inoculated. C2C12 myotubes infected with RML were collected at 4, 6, 8, 10, 13 and 15 days post-infection as in (A) and bioassayed. Infectivity of the C2C12 derived samples was calculated by comparing the observed incubation periods from each sample with those from brain homogenate dilutions and normalizing the data to protein content. Prion infectivity increases in C2C12 myotube cultures from day 4 to day 15 post-infection.

To determine the titre of myotube-generated PrP^Sc^, aliquots of cell homogenates were used to infect C57Bl/6 mice. To generate material for the bioassay, we infected T75 flasks of confluent myotubes with RML prions and harvested cells at indicated time points (Figure 3). 30 µL of this lysate was inoculated, intracerebrally, into weanling C57Bl/6 mice. A standard curve for infectivity was performed in parallel with mice being inoculated with serial 10-fold dilutions of RML brain homogenate (Figure S1). Mice inoculated with the infected C2C12 cell lysate (15 days post-infection) had an incubation period of 181.7 days. This suggests a level of infectivity approximate to a 0.3% brain homogenate from mice clinically affected with RML. Between 4- and 15- days post infection, the amount of infectivity present in C2C12 myotubes increased ∼10 fold and a statistically significant difference in incubation period was observed (Figure 3). C2C12 myotubes accumulated 10^5.5^ LD_50_/mg protein (Figure 3B) as calculated based upon comparison of incubation periods from C2C12 derived material compared to standard 10-fold dilutions of RML brain homogenate (Figure 3A). 10% RML brain homogenate contained 10^7^ LD_50_/mg protein (Figure 3B).

### PrP^Sc^ replication in myotubes does not cause any overt cellular pathology

**Figure 4:**
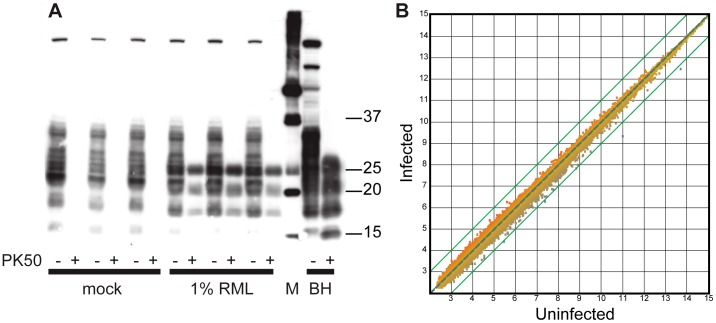
RML infection does not affect transcriptional profile of C2C12 myotubes. Gene expression profiling was performed from 3 replicate samples of uninfected and infected C2C12 myotube preparations. (A) Confirmation of PrP^Sc^ in cells used for gene expression profiling by immunoblotting with 3F10. Cell lysates were digested with 50 ug/mL of proteinase K (PK50) and 10 µg of protein was loaded in each lane. (B) Gene expression of infected vs. un-infected control cell cultures (N = 3). A scatter plot was created comparing the expression of genes in uninfected cells with that of infected cells. Each data point plotted is the average normalized signal intensity for each gene's expression (uninfected or infected). Data points outside the green lines have greater than 2-fold changes in gene expression. Transcription was globally unchanged in C2C12 cells replicating PrP^Sc^.

Cell cultures containing prion-infected and mock-infected myotubes were observed daily. Over the time course of the experiments (up to 21 days), no cell loss was observed in prion-infected plates as compared to mock-infected. To examine the cells for molecular responses to prion infection, gene expression profiling was performed (Figure 4). Expression profiles from infected and uninfected cells were similar and no genes were up-regulated greater than 2-fold by prion infection. Two genes were downregulated by >2-fold by prion infection, Carbonic anhydrase 3 and 2310046K23Rik, an un-annotated transcript. No genes were differentially regulated to an 85% confidence interval as assessed by T-test and a FDR based approach [36]. Subsequently, we examined those genes which were up-regulated in C2C12 cells infected with prion disease by submitting the “top 20” genes to the Prion Disease Database (http://prion.systemsbiology.net/) [37], [38] for which annotations were known. The genes were up-regulated in infected tubes between 1.36 and 1.6 fold. Four of the genes in the “top 20 list” (Iigp1, tgfb1, ifit1, Aldh1l2) were found to be up-regulated in brains of prion-infected mice suggesting that the changes observed might contain a prion-specific signature.

### PrP^Sc^ levels in myotubes are 10 fold greater than in N2a neuroblastoma and SMB cells

**Figure 5:**
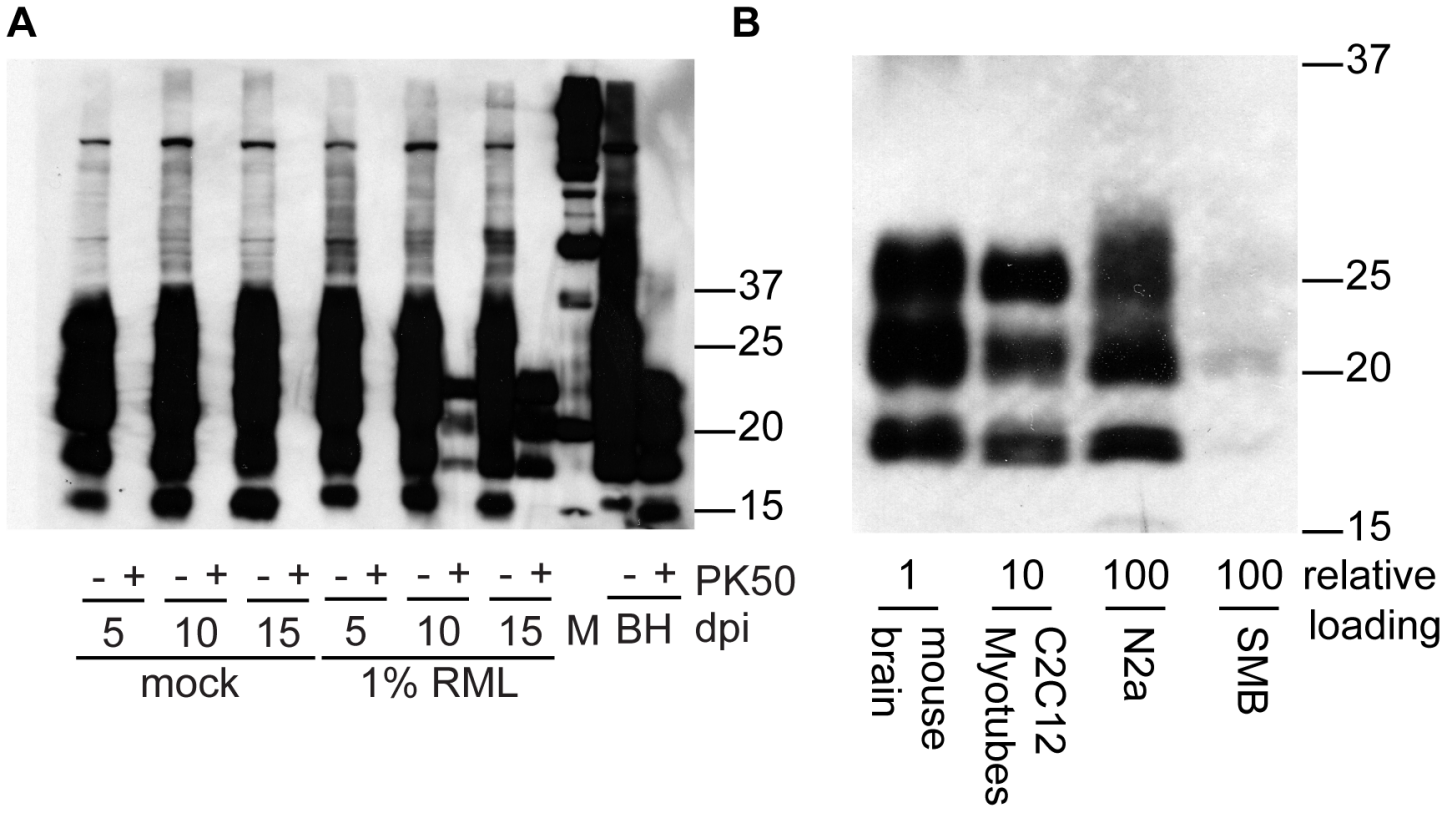
Accumulation of PrP^Sc^ in C2C12 myotubes, N2a neuroblastoma cells and SMB scrapie mouse brain cells. (A) Time dependent increase in PrP^Sc^ in C2C12 cells. 10 µg of protein (PK−) or protein equivalent (PK+) was loaded in each lane. (B) Relative abundance of PrP^Sc^ in clinically affected mouse brain homogenate and lysates from cell lines (C2C12 myotubes, N2a neuroblastoma cells and SMB scrapie mouse brain cells). All samples are treated with PK (50 µg/mL, final concentration). Relative loading is equivalent to protein amounts prior to proteinase K digestion 0.75 µg, 7.5 µg and 75 µg respectively. Antibody SAF 83 was used.

To compare PrP^Sc^ levels in prion-susceptible mouse cell lines, following exposure to RML agent, total protein (100 µg) collected from infected cell lysates and from infected brain homogenates was subjected to PK digestion (50 µg/mL final concentration). Strong signals were observed in the prion-infected C2C12 samples and the brain homogenates. Weaker PrP^Sc^ levels were observed in the N2a and SMB cells (Figure 5). To semi-quantify the differences in signal intensity, we digested and loaded ten times more total protein from infected myotubes than RML brain homogenate and ten times more N2a and SMB cell protein than myotube protein. These data indicate the PrP^Sc^ generation in C2C12 cells is considerably more robust than the other cell culture systems and within an order of magnitude of RML-infected brain homogenates.

### Pentosan polysulfate inhibits myotube prion replication

**Figure 6:**
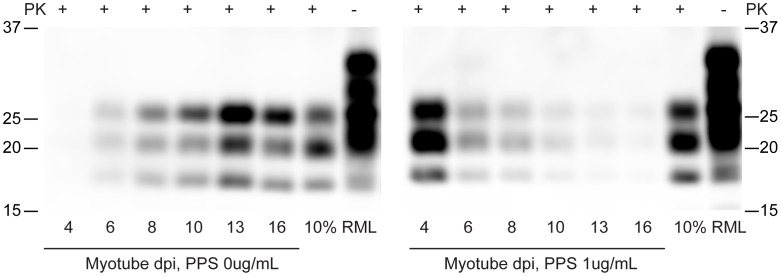
Pentosan polysulfate inhibits PrP^Sc^ accumulation in C2C12 myotubes. C2C12 cells were infected with 100 µL of 10% brain homogenates. Two days post infection, pentosan polysulfate (PPS) (1 µg/ml final concentration) was added to the media. PPS was kept on the cells until harvested at the designated dates. Antibody SAF 83 was used for western blot detection.

To examine whether known inhibitors of PrP^Sc^ replication could be used to “cure” prion-infected myotubes, we applied pentosan polysulfate to infected C2C12 myotubes. PPS is a sulfated polyanion previously identified to inhibit prion accumulation in cells [39], [40], prevent scrapie following intraperitoneal inoculation [41] and some therapeutic effect has been observed in human prion diseases, reviewed by Rainov et al., 2007 [42]. In our studies, replicate infected myotube cultures were treated with or without PPS (1 µg/mL final concentration) and harvested at specific time points. Equal amounts of total protein were subjected to PK digestion and immunoblotting (Figure 6). Without PPS, PrP^Sc^ abundance increased with time, whereas in cells treated with PPS, PrP^Sc^ accumulation was inhibited and the PrP^Sc^ signal substantially diminished by 16 days post-infection.

### C2C12 myotubes replicate 22L, ME7 and RML prions

**Figure 7:**
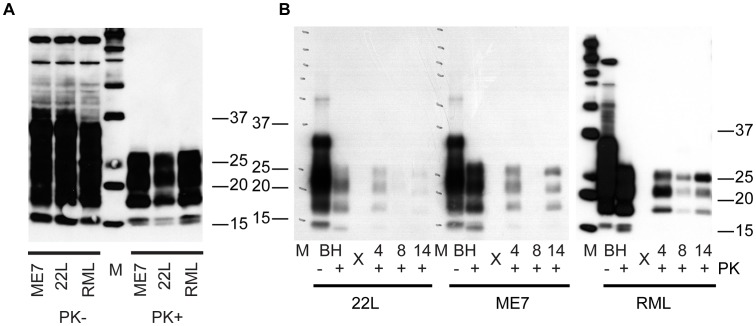
C2C12 myotubes are also susceptible to 22L and ME7 prions. (A) Brain homogenates (BH) from mice clinically affected with prion disease. 10 ul of 1% BH treated with or without 50 µg/ml PK was loaded in each lane. 22L and ME7 prions were generated in tga20 mice. RML prions were from C57Bl/6 mice. PrP^Sc^ was detected with 3F10 antibody. (B) C2C12 cells were infected with brain homogenates from 22L, ME7 and RML. At 4, 8 and 14 days post exposure, cells were lysed, lysates treated with 50 ug/mL of proteinase K and immunoblotted to detect the presence of PrP^Sc^. 30 µg of protein equivalent was loaded into each lane for 22L and ME7 samples. 10 µl of 0.1% BH was loaded as a control. 10 µg of protein equivalent from RML infected myotubes was loaded.

To examine the strain selectivity of the C2C12 myotube system, we infected differentiated myotube cultures with 3 different mouse prion strains (RML, 22L and ME7). Myotube cultures were infected in parallel and analyzed by immunoblotting for the presence of PrP^Sc^. All strains examined replicated prions in this myotube system (Figure 7), albeit with apparent strain specific kinetics. Signal at day 4 is carryover from the infection. The absence of signal at day 8 in 22L and ME7 would suggest that these strains replicate slower than RML, and the weak signal at day 14 in 22L would suggest that this strain replicates the slowest in C2C12 myotubes. The characteristic shift in glycosylation pattern to C2C12-like diglycosylated PrP is apparent.

### Prion replication is species-specific

**Figure 8:**
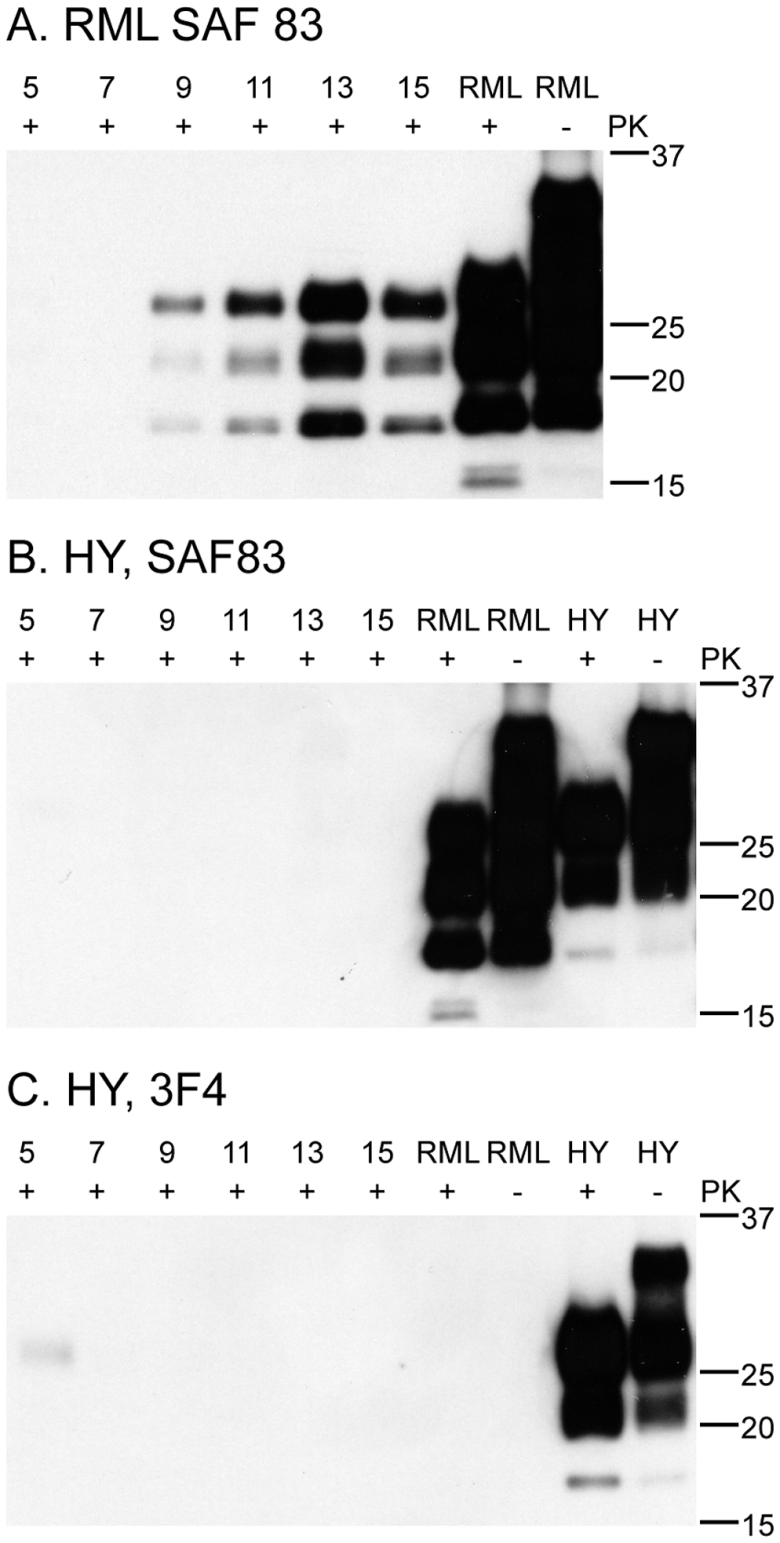
C2C12 myotubes replicate mouse RML prions, but not hamster HY prions. 15 µg of protein was loaded in each lane. RML and HY brain homogenates were 15 µl of 0.1% (A) C2C12 myotubes infected with RML accumulate PrP^Sc^ as detected by the SAF83 monoclonal antibody. SAF 83 recognizes mouse and hamster PrP. (B, C) C2C12 myotubes infected with hamster HY prions probed with SAF83 (B) and 3F4 (C) antibodies. 3F4 recognizes hamster PrP but not mouse, including some HY that persists in culture until day five. SAF83 also recognizes mouse prions, but no signal is detected at day 5. This is likely due to the higher affinity of 3F4 vs. the SAF83 antibody.

*In vivo* transmission of prions between different species is typically associated with a species barrier characterized by low penetrance and extended incubation period upon first passage. To examine the C2C12 model as an *in vitro* surrogate for examining interspecies transmission, we exposed the C2C12 myotubes to the Hyper (HY) strain of hamster-adapted transmissible mink encephalopathy prions at >10^9^ LD_50_/mL (Figure 8). While hamster PrP^HY^ appeared to persist in C2C12 myotube culture for 5 days, no mouse PrP^Sc^ was detected as a result of infection with HY prions. Control experiments using RML prions readily established PrP^Sc^ accumulation. In similar experiments using cervid prions, no conversion of mouse PrP^C^ was observed (data not shown).

## Discussion

We have demonstrated that non-replicative myotube cultures can readily be infected with mouse prion agent. PrP^Sc^ replication occurs in a short period of time, is robust, and levels of infectivity are relatively high. Input mouse brain homogenate-derived PrP^Sc^, evidenced by dominance of the mono-glycosylated band, routinely became undetectable after 5 days post-exposure (Figures 2, 5, 6, 7, 8). As media was changed daily, it is remains unclear whether the input PrP^Sc^ was actively degraded by the cells or diluted out by media changes. Three lines of evidence suggest that the C2C12 myotubes are replicating prions: 1) in scrapie mouse brain homogenate, the inocula used on our cells, the dominant band of PrP^Sc^ is monoglycosylated, whereas the C2C12-generated PrP^Sc^ is predominantly diglycosylated (Figures 2, 5, 6, 7, 8). The shift in the glycosylation pattern allows us to discriminate between input material and de novo generated material (Figure S2); 2) HY TME prions were only detectable at 5 days post-exposure suggesting agent does not persistence (Figure 8); and 3) bioassay of cell lysates demonstrated an increase in infectivity over the course of the cell infection (Figure 3). Therefore, we conclude that the PrP^Sc^ observed is due to replication and not persistence.

Although this is the first study to establish prion infection in C2C12 myotubes, we are not the first to attempt infection of C2C12 cells. Dlakic et al. established infections of C2C12 cells with 22L prions but only when co-cultured with susceptible N2a cells [34]. We were unsuccessful in infecting myoblasts but consistently infected myotubes directly, i.e., without co-infection with N2a cells. The successful infection of myotubes with 22L, ME7 and RML suggests that the differences observed are a result of both prion strain used for infection and/or the differentiation state of the C2C12 cells. Infection of myotubes with RML and ME7 was more robust than with 22L and we were only successful in infecting completely differentiated myotubes.

Typical proliferative cell cultures are constantly dividing and must be split. This means that, for prion infections to be maintained in culture, PrP^Sc^ replication must outpace cell replication and degradation [28], [43]. This is especially important considering that cell lines replicate at different rates. We observe that C2C12 myoblasts double every 18 hours. Contrast this cellular replication rate with *in vivo* accumulation (replication and degradation) of prions, a process that can take years to fulminate in clinical disease. The replication rate of cell lines may explain the differential susceptibility of different cell lines [11], [22], [24], [44]–[47] or clonally-derived sub-lines [24] to infection with different prion strains. In C2C12 cells, we observed replication of 3 different strains of prions, albeit with different levels of PrP^Sc^ at 14 days post-exposure. Another issue with proliferating cell lines is the potential genomic instability of these cells. For example, the median chromosome number of the N2a neuroblastoma cell line is 95 and the range is 59–193 (ATCC datasheet, CCL-131). By contrast, C2C12 myoblasts are diploid [48]. It is clear that stochastic genetic drift of chromosome number over generations of cell culture could cause changes in cell replication rate and/or PrP^C^ expression, both of which may affect PrP^Sc^ accumulation or molecular responses [35], [49]. Finally, proliferative adherent cell cultures are routinely passaged by trypsinization. Since PrP^C^ is expressed on the cell surface, and cleaved by trypsinization, passaging cells may result in a temporary decrease of PrP^C^ required for conversion. Myotube cultures, by contrast, are relatively static allowing direct comparison between parallel plates where the influence of cell division (replication rate, changes in chromosomal abnormalities, effect of trypsinization, etc) is removed.

Myotubes can accumulate substantial PrP^Sc^ (Figure 5) and associated infectivity (Figure 3). We routinely observe robust PrP^Sc^ signals from infected myotubes when loading only 10 µg of PK-digested total protein equivalent. Our data suggest that ∼10 fold higher levels of PrP^Sc^ are being generated in C2C12 myotube culture than in common N2a cells. While we observed substantially higher levels of PrP^Sc^ in C2C12 myotubes compared with chronically infected N2a cells, the heterogeneity of N2a cells with respect to PrP^Sc^ replication cautions against concluding with respect to PrP^Sc^ levels in general. Many different sublines of N2a cells can be isolated some which fail to replicate PrP^Sc^ and others which are quite prolific. Subclones of infected N2a cells accumulate 1 LD_50_ per 158 cells [50] or per 500 cells [21], suggesting a titre of approximately10^4^ LD_50_ per 1×10^7^ cells. A T75 flask of C2C12 myotubes at 15 days post infection contains 10^6^ LD_50_; as C2C12 myotubes form a monolayer of non-proliferative multinucleated myofibers, titre cannot be expressed as LD_50_ per cell, however, a confluent 75 cm^2^ flask of non-differentiated C2C12 myoblasts contains ∼1×10^7^ cells. This result is comparable to that observed in differentiated PC12 cells [44], [51] suggesting a similar conclusion, that differentiated non-replicative cells can accumulate prion infectivity to high levels. Importantly, C2C12 myotubes are not of neuronal origin, implying that the non-replicative differentiated state, and not the neuronal state, is the critical factor for allowing high levels of infectivity to accumulate in both C2C12 and PC12 cells.

We did not detect overt effects of prion infection in C2C12 myotubes as assessed by cell morphology or gene expression profiling despite the accumulation of appreciable levels of PrP^Sc^ and infectivity. This apparent lack of cell toxicity has been observed in most cell cultures systems [49] for *ex-vivo* PrP^Sc^ replication, excepting GT-1 cells [18]. The accumulation of high levels of infectivity in C2C12 myotubes and PC12 cells absent any observable cytopathic effect [51] suggests that PrP^Sc^ itself is generally non-toxic to cultured cells; the cytoxicity appears to be specific to neurons in the central nervous system [52]. This strongly suggests that the apparent lethality of prion disease must be due at least in part to the multiple cell types or complex architecture present in the CNS.

To examine the potential of myotubes to serve as a model system for assay of prion inhibition, we tested pentosan polysulfate (PPS), a well-established molecule with known anti-PrP^Sc^ replication properties, for its ability to hinder PrP^Sc^ accumulation in the myotubes. PPS inhibited PrP^Sc^ replication in the myotubes (Figure 6) but its efficacy was less than that observed in proliferative systems where PPS treatment and cell passage can completely remove infectivity [39], [40]. It is likely that, in cultured replicating cells, the dilutive effect of cell passage coupled with the PPS inhibition of PrP^Sc^ replication enhances the apparent effect of PPS. This observation supports the suggestion by Weissmann et al. [6] of that current proliferative cell-based models for assay of inhibition of PrP^Sc^ replication are inadequate. *In vivo*, there is no comparable dilution of PrP^Sc^ to that created by the serial passage of proliferative cell cultures. Treatment of infected myotubes with PPS also extended the time that inocula, as indicated by dominance of mono-glycosylated PrP^Sc^, persisted in the culture. A strong signal from carry-over inocula is present at day 4 in PPS treated cells and seems to persist longer (Figure 6b) than in myotubes without PPS (Figure 6a). One possibility is that PPS enhances the binding of PrP^Sc^ to cells preventing it being washed out by media changes. Alternatively, PPS may interfere with degradation of PrP^Sc^.

*In vivo*, transmission of hamster HY or white-tailed deer CWD prions to mice is not efficacious and is associated with a large species barrier[53], [54]. We examined our cell-based assay for prion replication fidelity by challenging C2C12 myotubes with hamster (Figure 8) and deer prions and were unable to replicate prions from either species, consistent with the normal host range of these agents. This suggests that C2C12 myotubes may be a good model system with which to probe species barriers and prion adaptation, at least with regard to mouse prion protein.

In summary, we have developed a novel, non-proliferative cell culture system that replicates prion infectivity to high levels generating substantial amounts of protease-resistant prion protein. This approach may be useful for probing prion strain and species barrier phenomena. Moreover, this system may allow a better assessment of putative anti-scrapie compounds as it removes the confounding effect of cell-replication from that of prion replication. We are currently adapting the myotube system for use with other prion agents.

## Materials and Methods

### Ethics statement

This study was carried out in accordance with the recommendations and guidelines of the Canadian Council on Animal Care under protocol 647/10/11C and was approved by the Institutional Animal Care Committee at the University of Alberta.

### Animal bioassay

All animal manipulation and care was performed under institutionally approved animal use protocols approved by the University of Alberta Animal Care and Use Committee. Preparations for bioassay were formulated/diluted in sterile water. 30 µL of each preparation was injected into the anterior fontanelle of weanling C57Bl/6 mice. Animals were scored weekly for the onset of clinical disease.

### Prions

Prion preparations were obtained by homogenization of brain tissue in Dulbecco's phosphate buffer or water. Brains were collected from animals afflicted with end-stage clinical prion disease, infection was confirmed by proteinase K digestion and western blotting. A 10% (w/v) homogenate prepared from a pool of RML affected brains was used for this work. This pooled homogenate was minimally 10^9^ LD_50_ per gram of brain as determined by bioassay using the Kärber formula [55]. Infectivity of C2C12 samples was calculated from the regression equation (Figure S1) derived from plotting the incubation period observed from inoculation of standard 10-fold dilutions of RML brain homogenates versus infectivity; time interval assay [56]. 22L and ME7 prions were prepared from clinically-affected tga20 mice [57] and contain approximately 10^8^ LD_50_ per mL [58].

### Cell culture

C2C12 (CRL-1722) myoblast cells were purchased from the American Type Culture Collection, expanded and aliquots stored in liquid nitrogen and expanded as needed. Myoblasts were grown in Dulbecco's Modified Eagle Medium, 10% fetal bovine serum (FBS) with penicillin and streptomycin (PS). Myoblasts were seeded onto experimental plates, differentiated when confluent by switching to differentiation medium; DMEM, 10% horse serum (HS), and PS. Three days after myotubes first appeared, infections were initiated by the addition of prion-infected brain homogenates diluted 1∶100 in differentiation media (DMEM, 1% HS, 1% PS). A typical infection experiment involved treatment of ∼1×10^7^ cells with ∼1×10^7^ LD_50_ infectious prions, 100 µL of 10% brain homogenate per 10 mL of media. See Methods S1. Media was changed daily, washing cells and removing residual inocula.

### Sample preparation and immunoblotting

Cell lysates were prepared by removing media, washing the cells with PBS and then adding RIPA lysis buffer. Total protein concentration was determined by BCA assay. Typically, 100 µg of total protein was digested with 3.5 µg of Proteinase K (PK) (Roche) for 30 minutes at 37°C in a volume of 70 uL (50 mg/ml PK final concentration). Digestion was terminated by addition of 30 ul of 5X SDS sample buffer. Each sample was loaded (10–15 µg based on the pre-digestion concentration) and resolved on 15-well 12% NuPAGE bis-Tris gels (Invitrogen), transferred to PVDF membrane and probed with anti-PrP antibodies 3F4 (a kind gift from Richard Rubenstein) epitope (107–112) [59], 3F10 (a kind gift from Yong-Sun Kim) epitope (137–151) [60] and/or SAF-83 (Cayman Chemical) epitope (126–164) [61]. Relative quantification of western blotting was performed by loading dilutions of samples until quasi-equivalent signals were obtained on western blots as determined by image analysis software (Adobe Photoshop).

### Gene expression profiling

Gene expression profiling was performed as described [62]. Briefly, RNA was purified from cell pellets using the QIAshredder and RNeasy mini kit (Qiagen, Valencia, CA) in accordance with the manufacturers' instructions. Total RNA was amplified and labeled in preparation for chemical fragmentation and hybridization with the MessageAmp Premier RNA amplification and labeling kit (Life Technologies, Grand Island, NY). Amplified and labelled cRNAs were hybridized on Affymetrix (Santa Clara, CA) mouse genome 430 2.0 high density oligonucleotide arrays. Raw data were analyzed with Arraystar 5.0 (DNA Star, Madison, WI). Robust multiarray normalization using the quantile approach was used to normalize all microarray data. Data are deposited into the National Center for Biotechnology Information Gene expression omnibus database with accession number GSE44563.

### Accession numbers of genes or protein listed in the text

Prion Protein (MGI:97769), 2310046K23Rik (MGI:1924218),Carbonic Anhydrase 3(MGI:88270), Iigp1 (MGI:1926259), tgfb1(MGI:98725), ifit1 (MGI:99450), Aldh1l2(MGI:2444680)

## Supporting Information

Figure S1 Time interval assay for determination of infectivity present in experimental inocula. The infectivity (as determined by end-point dilution) present in a 10-fold dilution series of RML infected brain homogenate was plotted against the incubation period observed for each dilution and the regression equation determined: Log (L.D.50) = −8.77(Observed incubation period)+234.27. R2 = 0.8.
(TIF)

Figure S2 Analysis of glycosylation pattern from C2C12 myotube and Brain derived PrP^Sc^. Relative abundances of un-, mono- and di-glycosylated bands were calculated as a percent of the total PrPSc signal. A statistically significant difference (P<0.05) was observed in the abundances of mono- and di-glycosylated PrPSc derived from C2C12 myotubes and mouse brain tissue.
(TIF)

Methods S1 Detailed protocol for culture of C2C12 myoblasts, differentiation into myotubes and infection with prion agents.
(DOCX)
