# Supplementary material for: Infectious Prions Accumulate to High Levels in Non Proliferative C2C12 Myotubes
Source: PLoS Pathog. 2013 Nov 7;9(11):e1003755. doi: 10.1371/journal.ppat.1003755 (PMC3820720; doi:10.1371/journal.ppat.1003755)
Supplement: Methods S1 — Detailed protocol for culture of C2C12 myoblasts, differentiation into myotubes and infection with prion agents. (DOCX) [file ppat.1003755.s003.docx]

**C2C12 myoblast cell culture**

C2C12 myoblasts (ATCC CRL-1772) are grown in Dulbecco's Modified Eagle Media (DMEM) (Gibco cat# 11995-073) containing 25mM glucose and 1mM pyruvate. DMEM is supplemented with 10% fetal bovine serum (FBS) and Penicillin G + Streptomycin (PS). C2C12 myoblasts double every 18 hours. Cells should not reach confluence unless they are to be differentiated. Myoblasts can be subcultured by trypsinization (described in ATCC protocol) and expanded until a suitable number of cells are prepared for the experiment. Cells are maintained in liquid N2 (Passages 4-6) and expanded from stock as necessary for experiments.

6-well plates are routinely used for infection experiments. Each well on the plate is infected in parallel and separate 6-well plates are used for different treatments. Avoid culturing infected and uninfected cells within the same plate. To prepare C2C12 myotubes for an infection, seed C2C12 myoblasts at low density <10,000 cells /cm^2^. Myotubes adhere better when they are allowed to expand to confluence on the plate and plating myoblasts at a high density can result in loss of the myotube layer following differentiation.

C2C12 myoblast culture media C2C12 myotube culture media

DMEM 500mL DMEM 500mL

PS 5mL PS 5mL

**FBS 50mL HS 50mL**

**Differentiating C2C12 myoblasts, infecting and maintaining myotube cultures**

Once the myoblasts have reached confluence, exchange the myoblast media with myotube media containing 10% horse serum (HS). In 2-3 days, myotubes will begin to appear. After 4 days, the myoblasts will have completely formed myotubes. To infect the cells, prepare an infection media by adding brain homogenate directly to the myotube culture media in a ratio of 100:1. For example, add 120µl of 10% brain homogenate to 12mL of media and mix completely. This media can then be applied to the cells to infect, 2ml per well in a 6-well plate. Lower dilutions of brain homogenate can be used, however, decreasing the amount of HS in the infection media to 1% is recommended. On the following days, exchange the media with fresh pre-warmed myotube culture media daily to avoid acidification of the media. Plates containing infected cells should always be processed from least infectivity to most to prevent cross-contamination.

**Collecting cell lysate for PrP^Sc^ assay**

To collect myotubes, aspirate the media, and wash the monolayer with Dulbecco's PBS (Gibco cat#14190-144). Aspirate all PBS and add RIPA lysis buffer containing protease inhibitors, approximately ~500µL of RIPA lysis buffer per well on a 6 well plate. Allow the lysis buffer to rest on the cell layer for a minute at which time it should be possible to collect the entire cell layer. If the cell layer sticks, aspirate the lysis buffer working from one edge of the well, until the whole sheet of cells can be collected. After collection, gently pipet the cell layer up and down in the microfuge tube to disperse the cells. After brief sonication, the lysate can be used for protein assay, proteinase K (PK) digestion and immunoblotting. For PK digestion reactions, 100µg of total protein is digested with 3.5µg of PK in a final volume of 70µL (50µg/mL). Best results are obtained with Roche recombinant PCR-grade PK (cat# 03115828001) in solution, which has a standardized activity of 50 or 600 Units/mL in the chromozyme and hemoglobin assays, respectively, to ensure consistent reproducible PK digestions.
